# Case report: A mesenchymal chondrosarcoma with alternative *HEY1::NCOA2* fusions in the sella turcica

**DOI:** 10.3389/pore.2024.1611730

**Published:** 2024-08-06

**Authors:** Satsuki Kishikawa, Akihide Kondo, Takashi Yao, Tsuyoshi Saito

**Affiliations:** ^1^ Department of Human Pathology, Graduate School of Medicine, Juntendo University, Tokyo, Japan; ^2^ Department of Neurosurgery, Graduate School of Medicine, Juntendo University, Tokyo, Japan

**Keywords:** mesenchymal chondrosarcoma, intracranial mesenchymal chondrosarcoma, CNS sarcoma, *HEY1::NCOA2*, sella turcica

## Abstract

**Introduction:**

Mesenchymal chondrosarcoma (MCS) is a rare subtype of chondrosarcoma that occurs at widespread anatomical locations, such as bone, soft tissue, and intracranial sites. The central nervous system (CNS) is one of the most common origins of extraosseous MCS. However, alternative *HEY1::NCOA2* fusions have not been reported in this tumor.

**Case report:**

We report a case of intracranial MCS with *HEY1::NCOA2* rearrangement. A 52-year-old woman presented with a 15-mm calcified mass around the sella turcica. She initially underwent transsphenoidal surgery for tumor resection and then additional resections for five local recurrences over 5 years. Histologically, the tumor was composed of small round to spindle-shaped cells admixed with well-differentiated hyaline cartilaginous islands. A hemangiopericytoma-like vascular pattern and small sinusoid-like vessels were also observed. RNA sequencing using RNA extracted from formalin-fixed paraffin-embedded samples from the last operation revealed two alternative variants of the *HEY1::NCOA2* fusion: *HEY1*(ex4)::*NCOA2* (ex13) and *HEY1*(ex4)::*NCOA2*(ex14). Both variants were confirmed as in-frame fusions using reverse transcription-polymerase chain reaction.

**Discussion:**

Cartilaginous components were often not apparent during the recurrences. In addition to the non-typical pathological finding, the correct diagnosis was hampered by the poor RNA quality of the surgical specimens and non-specific STAT6 nuclear staining.

**Conclusion:**

This is the first reported case of intracranial MCS with an alternative *HEY1::NCOA2* fusion.

## Introduction

Mesenchymal chondrosarcoma (MCS) is a rare subtype of chondrosarcoma, accounting for only 2%–4% of all chondrosarcomas [1]. MCS is distributed in the bone, soft tissue, and intracranial sites. Although the meninges are one of the most common extraskeletal origins of MCS [2], intracranial MCS is an extremely rare tumor of the central nervous system (CNS). Intracranial MCS usually occurs in adolescents and young adults and accounts for less than 1% of all intracranial tumors [3]. When limited to sarcomas, MCS accounts for 11.5% of all CNS sarcomas [4]. One of the most frequent sites for MCSs is the head and neck region (which contains bones in addition to the CNS), accounting for 13% of MCSs [5]. In 2012, *HEY1::NCOA2* fusion was identified in MCS [6]. Subsequently, *IRF2BP2::CDX1* fusion was detected [7]. In 2020, NKX3.1 expression was reported as a useful immunohistochemical marker for MCS [8, 9]. By identifying such fusion genes or confirming NKX3.1 expression by immunohistochemistry (IHC), it is easier to reach an accurate diagnosis of MCS. MCS is still a very rare tumor, and it goes without saying that listing MCS in the differential diagnoses is important to perform the above diagnostic utilities. We describe a case of intracranial MCS harboring alternative variants of the *HEY1::NCOA2* fusion gene in a 52-year-old woman.

## Case report

### Clinical case

**FIGURE 1:**
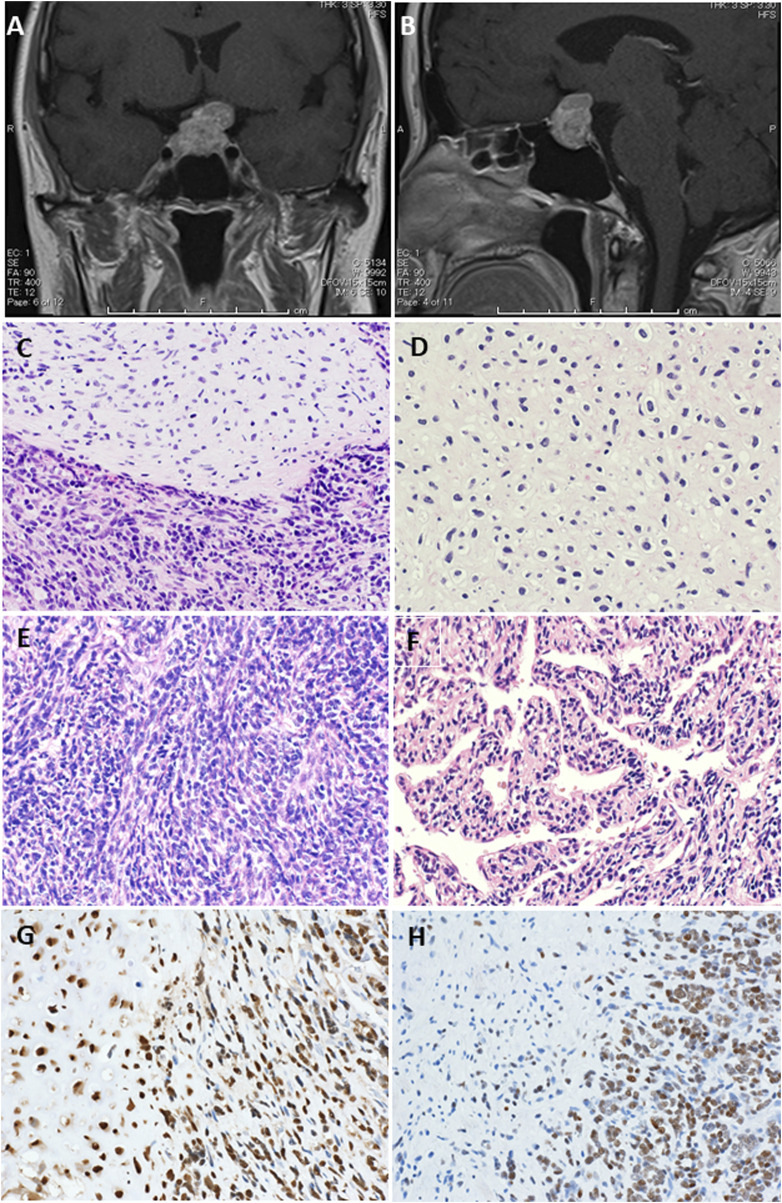
Magnetic resonance imaging (MRI) with contrast enhancement. Preoperative MRI showed a protruding mass above the sella turcica [**(A)** coronal view, **(B)** sagittal view]. **(C–F)** Representative pathological findings. Hematoxylin-eosin staining showed a tumor composed of small round cells and islands of well-differentiated hyaline cartilage with atypical chondroid cells floating in lacunar spaces (original magnification, ×200) **(C)**. A neoplastic cartilage cells do not show higher nuclear atypia, but they have constricted nuclei (original magnification, ×200) **(D)**. Areas of dense proliferation of spindle cells are also encountered (original magnification, ×200) **(E)**. A hemangiopericytomatous proliferation pattern is also noted (original magnification, ×200) **(F)**. SOX9 immunohistochemistry shows positive staining in both spindle cell and cartilaginous areas, whereas NKX3.1 immunohistochemistry shows positive staining almost only in spindle cell area (original magnification, ×200, each) **(G)**.

A 52-year-old Japanese woman initially noticed haze in her left eye. She was referred to our hospital because she subsequently showed gradual exacerbation of bitemporal hemianopia. She and her family had no specific medical history. Computed tomography of the head revealed a 15-mm calcified mass in the sella turcica. Magnetic resonance imaging (MRI) of the brain revealed a mass protruding over the sella turcica, with the normal pituitary gland pressed to the upper right and the optic chiasm pressed to the upper left (Figures 1A, B).

### Surgery

Transsphenoidal surgery was performed, but unfortunately it was incomplete resection. The pathological diagnosis was limited to malignant tumor, with differential diagnoses of solitary fibrous tumor (SFT) and MCS. Ten months later, MRI revealed tumor recurrence. The relapsed tumor was resected via craniotomy, and the patient received stereotactic radiotherapy (SRT). Four years after SRT, she underwent four of surgical resections and one SRT for several local recurrences.

### Histopathological findings

**FIGURE 2:**
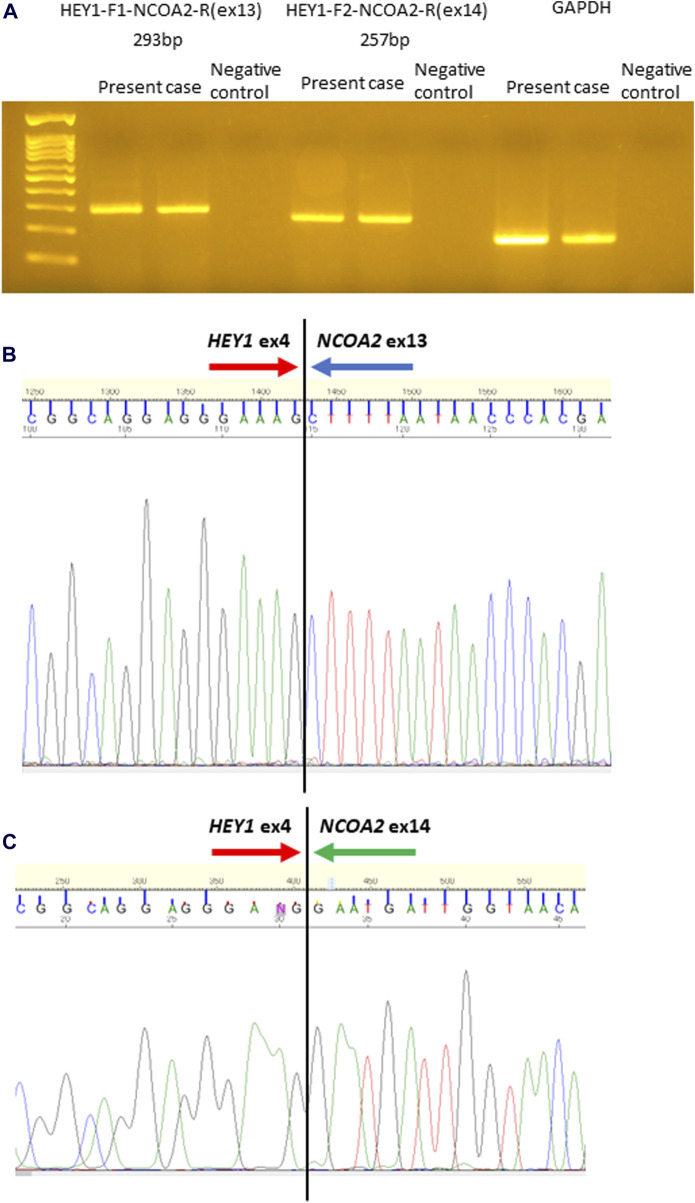
Identification of *HEY1::NCOA2* fusion using reverse transcription-polymerase chain reaction (RT-PCR) followed by direct sequencing. RT-PCR was performed using *HEY1*(ex4)::*NCOA2*(ex13) fusion- and *HEY1*(ex4)::*NCOA2*(ex14) fusion-specific primer pairs. Anticipated sizes for each RT-PCR product were 293 and 257 bp, respectively **(A)**. Sequence analysis demonstrated that exon 4 of *HEY1* was fused to exon 13 of *NCOA2* **(B)** and exon 14 of *NCOA2* **(C)**.

Histologically, the tumor was mainly composed of small round cells, and some well-differentiated hyaline cartilage islands with atypical chondroid cells floating in the lacunar spaces were also observed. The cellularity of the atypical chondroid cells was high. No ossification or calcification within the cartilaginous area was observed. In some places, the small cells exhibited a spindle-shaped morphology. A hemangiopericytomatous proliferation pattern was also noted (Figures 1C–F). No meningothelial architecture was observed. Immunohistochemical examinations were performed using antibodies against CD99 (12E7; Dako, Santa Clara, CA, United States), S100 (polyclonal; Dako), STAT6 (YE361; Abcam, Cambridge, United Kingdom), CD34 (QBEnd10; Dako), EMA (E29; Dako), cytokeratin AE1/AE3 (AE1/AE3; Dako), CAM5.2 (CAM5.2; BD Biosciences), SOX9 (EPR14335; Abcam), NKX3.1 (D6D2Z; Cell Signaling), p53 (PAB1801; Thermo Fisher Scientific), and Ki-67 (MIB-1; Dako) following the manufacturer’s instructions. Immunohistochemically, the small round to spindle-shaped cells were positive for CD99 but negative for STAT6, CD34, EMA, and AE1/AE3, whereas the cartilaginous lesions were focally positive for S100. The tumor cells showed focal staining for CAM5.2, but p53 overexpression was not evident. The Ki-67 labeling index was approximately 50%. Then, RNA sequencing (Riken Genesis Co., Ltd., Kanagawa, Japan) was performed using RNA from FFPE samples from the last operation, and two subtypes of *HEY1*::*NCOA2* fusion were identified: fused *HEY1* ex4 to *NCOA2* ex13 and fused *HEY1* ex4 to *NCOA2* ex14. RNA sequencing revealed that read counts for *HEY1* (ex4)::*NCOA2* (ex13) fusion and *HEY1* (ex4)::*NCOA2* (ex14) fusion were 566 and 172, respectively. Both alterations were confirmed as in-frame fusions by RT-PCR using the following primer pairs: 5′-ACC​GGA​TCA​ATA​ACA​GTT​TG-3′ (HEY1-F1) and 5′-GTG​ATA​CCT​CAG​CCA​GGA-3′ (NCOA2-R1) and 5′-CCG​AGA​TCC​TGC​AGA​TGA​CC-3′ (HEY1-F2) and 5′-GCC​AAA​GAC​AGA​CGC​TTC​AG-3′ (NCOA2-R2) (Figures 2A–C). Finally, this case was diagnosed as mesenchymal chondrosarcoma. Then, additional immunohistochemical examinations were performed, and positive stainings for SOX9 and NKX3.1 were confirmed (Figures 1G, H).

### Follow up

After several times of surgery, the patient experienced blindness and hydrocephalus due to invasion of the recurrent tumors. Regarding the additional therapy to this patient, chemotherapy was not performed, because resistance to chemotherapy and radiation therapy has been reported in conventional chondrosarcoma and it is still controversial in mesenchymal chondrosarcoma [10–13].

## Discussion

At the initial resection, characteristic pathological findings of small round cells admixed with well-differentiated hyaline cartilaginous islands suggested MCS as a differential diagnosis. Tumor-specific *HEY1::NCOA2* fusion was not detected at that time, probably because of poor sample quality. In this case, we could not confirm the simultaneous presence of cartilaginous components and small round cell areas in several recurrent surgical specimens. In addition, undifferentiated small round cells frequently showed a spindle-shaped morphology with a hemangiopericytomatous vascular pattern. Furthermore, repeated STAT6 IHC revealed no nuclear staining which ruled out the possibility of an SFT.

Clinicopathologic characteristics of MCS in head and neck regions including brain origin was well described in a recent study [11]. It occurs in relatively younger age, and the median age at diagnosis was 19 years (range: 6–54 years) [11]. The patient in this case was oldest among 4 MCS of brain origin in that study [11]. Absence of cartilagenous area was observed in 4 of 13 cases [11] as seen in the recurrent tumor of our case.

Histologically, the tumor was composed of small round to spindle-shaped cells with a hemangiopericytomatous vasculature. Staghorn/hemangiopericytomatous vessels were also described as a frequent histological feature as seen in this case [11]. The differential diagnoses were Ewing’s sarcoma, synovial sarcoma, and SFT. It is difficult to correctly diagnose MCS, especially when only a small round cell area is collected, as was the case for the second and subsequent surgical specimens in this case. Immunostaining for NKX3.1 [8] and SOX9, a master regulator of chondrogenesis, has been reported to be useful [14]. IHC for NKX3.1 and SOX9 was also performed in this case. Tumor cells in both small round/spindle cell and cartilage areas showed positive staining for SOX9, while almost only small round/spindle cell area showed positive staining for NKX3.1. Thus, IHC using these antibodies may be able to distinguish tissues composed of only small round cells without cartilage components.

*HEY1*(ex4)::*NCOA2*(ex13) fusion has been reported as *HEY1*-*NCOA2* fusion in MCS; however, *HEY1*(ex4)::*NCOA2*(ex14) fusion has not been reported to date. The mechanism of generating the two alternative types of *HEY1*::*NCOA2* fusion in this case [*HEY1*(ex4)::*NCOA2*(ex13) fusion and *HEY1*(ex4)::*NCOA2*(ex14) fusion] is unknown, although *HEY1*(ex4)::*NCOA2*(ex13) fusion was approximately three times dominant than *HEY1*(ex4)::*NCOA2*(ex14) fusion. These two forms of *HEY1*::*NCOA2* fusion might be caused by splicing alterations, as an *EWSR1::ATF1* fusion was identified in clear cell sarcoma [15], although we do not have any data. Furthermore, it is not clear whether this short form of *HEY1::NCOA2* fusion lacking exon13 of *NCOA2* is oncogenic, since the functional study has not so far been performed.

Therapeutic strategies that are currently believed to reduce the risk of recurrence include radical resection with radiation and chemotherapy. Xu et al. reported that two patients with MCS who received neoadjuvant chemotherapy did not show any therapeutic response [11], and Huvos et al. also demonstrated that four patients with MCS did not show response to preoperative high dose methotrexate-based chemotherapy [12]. However, Tsuda et al. found stable disease in three MCS patients and partial response in one MCS patient treated with neoadjuvant chemotherapy [13]. Thus, chemotherapeutic effect on MCS is still controversial, and new therapeutic option is expected.

Regarding tumorigenesis, Qi et al. reported that platelet-derived growth factor receptor alpha, which belongs to a family of receptor tyrosine kinases, was upregulated by *HEY1::NCOA2* fusion in a study using transduced induced pluripotent stem cell MSCs with inducible expression of the *HEY1::NCOA2* fusion protein [16]. In addition, a recent report showed that imatinib, a tyrosine kinase inhibitor (TKI), significantly reduced tumor growth in the *HEY1::NCOA2* fusion-driven cellular model as well as in MCS-patient derived xenograft models [17]. Therefore, although further research is required, TKI may be effective in treating MCS.

## Conclusion

We encountered a case of intracranial MCS harboring two alternative forms of the *HEY1::NCOA2* fusion transcripts.

## Data Availability

The raw data supporting the conclusion of this article will be made available by the authors, without undue reservation.
